# Cardiac Efficiency as a Potential Marker of Firefighter Expertise: A Pilot Study of Heart Rate Variability Monitoring in Realistic Search‐and‐Rescue Training

**DOI:** 10.1155/tsm2/5788863

**Published:** 2026-10-11

**Authors:** Xia Zhong, Yu Yan, Haoxiang Gao, Jingyi Wang, Siyuan Tang, Guang Ge, Jinzhen Zhao

**Affiliations:** ^1^ Shanghai Fire Research Institute of Ministry of Emergency Management (MEM) Shanghai 200032 China; ^2^ Training and Service Support Detachment Shanghai Fire and Rescue Corps Shanghai 200336 China

**Keywords:** heart rate variability, occupational training, parasympathetic recovery, physiological efficiency, spatial navigation

## Abstract

Expertise in high‐stakes occupations is typically evaluated through performance outcomes, potentially neglecting physiological efficiency—a dimension critical for career longevity in physically demanding work. In this pilot study, we investigated whether expert firefighters achieve comparable task performance with lower cardiovascular activation and faster autonomic recovery than novices during realistic search‐and‐rescue operations. Twenty‐two male firefighters completed navigation tasks in a high‐fidelity training facility under operational conditions (complete darkness, full protective equipment). Using a 2 × 2 quasi‐experimental design, we manipulated navigation strategies (route‐based vs. survey‐based) and continuously recorded heart rate variability across baseline, execution, and recovery periods. Our preliminary data suggest that behavioral outcomes and physiological responses may be largely decoupled in this context. Experts and novices demonstrated equivalent task completion times and accuracy. However, experts exhibited substantially lower heart rate elevation during tasks and 46% greater parasympathetic recovery post‐task. Critically, an expertise‐by‐strategy interaction suggested that experts’ efficiency advantages were magnified when employing route‐based strategies aligned with training protocols, whereas novices showed no strategy‐dependent differences. These findings provide preliminary evidence that physiological efficiency represents a distinct expertise component, suggesting that identical overt performance can mask vastly different internal costs. Heart rate variability monitoring may therefore serve as a useful exploratory tool to help identify individuals requiring additional skill consolidation or recovery support before overt performance degrades.

## 1. Introduction

Expertise is traditionally defined by superior performance outcomes: experts solve problems faster, make fewer errors, and achieve better results than novices [1, 2]. However, this performance‐centric view may overlook a critical dimension of skilled behavior—the efficiency with which outcomes are achieved. Foundational psychophysiological research, such as the neural efficiency hypothesis [3], suggests that experts optimize their resource allocation, demonstrating lower cortical and autonomic activation while maintaining high performance. Recent theoretical frameworks further posit that true expertise may manifest not only in what is accomplished, but also in how efficiently it is accomplished, particularly in terms of physiological resource allocation [4, 5]. Theoretically, as individuals develop expertise, their reliance on effortful controlled cognition decreases while automated processing and emotion‐cognition integration improve, allowing them to manage task demands with reduced physiological costs. From this perspective, experts and novices might achieve similar task outcomes, yet differ profoundly in the underlying physiological costs required to sustain performance.

Firefighting exemplifies a high‐stakes occupation where physiological efficiency is paramount. Firefighters routinely operate in extreme environments—elevated temperatures, smoke‐obscured visibility, time pressure, and life‐threatening hazards—where cognitive and physical demands are substantial [6, 7]. Despite decades of research on firefighter performance, most studies have focused on physical fitness, task completion times, or decision‐making accuracy [8, 9], with relatively little attention to the physiological costs of cognitive operations during task execution. Excessive physiological strain—even when performance appears adequate—may compromise safety, accelerate fatigue, and impair decision‐making in prolonged operations [10]. Critically, optimizing physiological efficiency is directly linked to critical occupational safety outcomes, as it reduces cumulative allostatic load, preserves cognitive bandwidth for sudden emergency responses, and potentially mitigates the risk of long‐term cardiovascular morbidity. To examine these questions under ecologically valid conditions, the present study utilized a Compartment Fire Behavior Training (CFBT) facility that simulates realistic fire and smoke conditions while maintaining safety protocols [11], providing an ideal setting for assessing physiological responses during high‐fidelity search‐and‐rescue tasks.

Physiological measurement offers a unique window into the “hidden” costs of expertise that behavioral metrics alone cannot reveal. Importantly, physiological changes during complex tasks do not merely reflect physical exertion; they are robust indicators of continuous mental processes, stress coping, and self‐regulation. According to the neurovisceral integration model [12] and the vagal tank theory [13], cardiac measures are intrinsically linked to prefrontal cortical function, serving as an index of how effectively an individual integrates emotional regulation and cognitive control under stress. Crucially, within this theoretical framework, HRV serves as a dynamic marker of expertise rather than merely an indicator of general time‐served experience or baseline physiological conditioning. While cardiovascular fitness undoubtedly facilitates overall physical work capacity, task‐evoked HRV modulation and rapid post‐task recovery specifically index the expert’s superior cognitive‐emotional regulation. It reflects the ability to efficiently allocate cognitive resources, maintain automated processing, and regulate threat appraisal under pressure, independent of baseline physical fitness levels. Cardiac and pupillary responses reflect real‐time cognitive and autonomic processes during task execution [14, 15], while recovery capacity—an often‐overlooked dimension of occupational performance—becomes critical during extended operations or repeated exposures to demanding scenarios [16].

Spatial navigation during search‐and‐rescue operations provides an ideal cognitive task for examining expertise‐related differences in physiological efficiency. Navigation in complex environments imposes substantial demands on working memory, spatial attention, and executive control [17, 18]. Individuals can employ different spatial strategies: survey strategies involve constructing a mental representation of the overall spatial layout, whereas route strategies rely on procedural sequences of turns and landmarks [19, 20]. These strategies impose different cognitive loads: survey strategies require greater working memory for maintaining allocentric representations, while route strategies depend on sequential procedural memory [21, 22]. Spatial strategies can be systematically induced through task framing—encoding spatial locations for map‐based recall promotes survey strategies, whereas attending to sequential landmarks promotes route strategies [20]. This task‐based strategy induction offers higher ecological validity than explicit strategy instructions, as it allows strategies to emerge naturally from task demands rather than imposed constraints [23].

To assess physiological efficiency, the present study employed heart rate variability (HRV) and pupillary responses as complementary physiological indices. HRV, particularly time‐domain indices such as the root mean square of successive differences (RMSSD), reflects parasympathetic nervous system activity and has been established as a reliable indicator of cognitive load and self‐regulatory capacity [12, 13]. Higher HRV during demanding tasks and faster post‐task recovery are associated with greater cognitive efficiency and adaptive stress responses [24]. Pupillary responses were recorded as an exploratory measure of sympathetic activation and cognitive effort during task execution [25, 26].

Furthermore, the broader sensitivity and clinical utility of HRV extend well beyond evaluating momentary cognitive load, reflecting dynamic autonomic regulation and demonstrating broad clinical and translational applications [27]. HRV is profoundly sensitive to various physiological perturbations, including infectious and postinfectious states. For example, post‐COVID‐19 patients exhibit marked alterations in cardiac autonomic function, reflected by altered HRV indices [28]. Similarly, structured physical activities and exercise interventions also produce clinically meaningful changes in HRV. In the context of occupational and personalized health, HRV is increasingly incorporated into precision medicine as a noninvasive predictive biomarker for cardiovascular resilience and adverse outcomes. Recent studies have demonstrated the utility of mobile‐based HRV monitoring for tracking cardiovascular risk trajectories in high‐risk groups, such as diabetic patients [29]. Moreover, HRV‐derived nomograms have been successfully validated for predicting atrial fibrillation [30], and integrated into new risk prediction models for assessing myocardial injury in elderly patients undergoing nonelective surgery [31]. Acknowledging this growing translational importance underscores why HRV is a highly promising tool for noninvasive occupational monitoring, capable of facilitating individualized risk stratification and optimizing both performance and health trajectories in first responders. Specifically, recent applications of HRV monitoring in first responders—such as assessing autonomic reactivity during smoke chamber tests in firefighters [15] and evaluating stress resilience in tactical law enforcement personnel—further strengthen the rationale for using HRV as a sensitive, ecologically valid metric of operational expertise and cognitive‐emotional readiness in emergency scenarios.

Despite the theoretical and practical importance of physiological efficiency in understanding expertise, several critical gaps remain. First, most expertise research has examined either behavioral performance or physiological responses, but rarely both simultaneously in ecologically valid settings [32], making it difficult to distinguish whether experts exhibit lower physiological costs despite similar performance. Second, few studies have examined how expertise modulates physiological responses during complex, dynamic tasks that simulate real‐world occupational demands. Third, the potential interaction between expertise and task strategy—whether experts and novices differentially benefit from different spatial navigation strategies—remains underexplored, with direct implications for training design and operational protocols.

The present study addressed these gaps by comparing cardiac and pupillary responses between expert and novice firefighters during simulated search‐and‐rescue navigation tasks in a high‐fidelity CFBT facility. Spatial navigation strategies were manipulated through task‐framing procedures: route‐strategy participants attended to sequential landmarks (fluorescent markers) along the navigation path, whereas survey‐strategy participants encoded victim locations for subsequent map‐based recall [17, 21]. We measured cardiac and pupillary responses during task execution and post‐task HRV recovery, alongside behavioral performance (completion time and victims located), with participants randomly assigned to strategy conditions and expertise groups balanced across conditions. Given the relatively small sample size and the highly demanding operational environment, this pilot study aimed to provide preliminary data on these autonomic patterns.

We tentatively hypothesized that

 
**(H1a) Main effect of expertise on physiological costs:** Expert firefighters might exhibit lower physiological costs than novices during task execution, as evidenced by higher HRV and smaller task‐evoked pupil dilations within each strategy condition.
 
**(H1b) Main effect of expertise on recovery:** Experts would potentially demonstrate faster physiological recovery post‐task, reflected in more rapid return of HRV and pupil diameters to baseline levels.
 
**(H2) Main effect of strategy:** Participants in the survey‐strategy condition were expected to show higher physiological costs (lower HRV, larger pupil dilations) than those in the route‐strategy condition, reflecting the greater cognitive demands of maintaining allocentric spatial representations.
 
**(H3) Expertise × Strategy interaction:** The physiological cost differential between survey and route strategies would be smaller for experts than novices, indicating that expertise may confer strategic flexibility in managing differential cognitive demands.

## 2. Method

### 2.1. Participants

This study received institutional approval from the National Fire and Rescue Administration. All participants provided written informed consent prior to participation.

Twenty‐two male firefighters holding the operational position of “fire combatant” were recruited from fire brigades in China, and classified into two expertise groups based on operational experience (see Table 1). The all‐male sample reflects the operational structure of the Chinese National Fire and Rescue Service, where female personnel are primarily assigned to administrative, logistics, and support roles rather than frontline firefighting and rescue operations. Female personnel are not required to participate in firefighting training or operational deployments. This organizational structure is comparable to many fire and rescue services internationally, where frontline operational roles remain predominantly male‐dominated.

**TABLE 1:** Participant characteristics and distribution.

Characteristic	Novice firefighters	Expert firefighters	Total sample
Sample size (*n*)	10	12	22
Age (years)	21.40 ± 2.22	28.67 ± 4.44	25.36 ± 5.11
Service years	< 2	7.32 ± 2.94	—
Navigation strategy distribution			
Route strategy	5 (50%)	6 (50%)	11 (50%)
Survey strategy	5 (50%)	6 (50%)	11 (50%)
Operational position	Fire combatant		
Location	China		

*Note:* All values are presented as *M* ± SD unless otherwise specified. All participants were male firefighters engaged in frontline firefighting and search‐and‐rescue operations.

Novice firefighters (*n* = 10) were recruited and had completed basic training but had no real‐world emergency response experience. Expert firefighters (*n* = 12) were experienced personnel serving as instructors at the training academy, with substantial field experience in structural firefighting and rescue operations.

All participants self‐identified as Han Chinese, reflecting the demographic composition of the regional fire service. Educational background varied by expertise level: novice firefighters had completed high school or vocational training (12–13 years of formal education), while expert firefighters held high school diplomas with additional specialized firefighting certifications (*M* = 13.2 years of education, SD = 1.1). Socioeconomic status information was not collected due to institutional privacy policies; however, all participants were civil service employees with standardized compensation structures according to Chinese national firefighter standards. All participants met the physical fitness requirements specified in the Chinese national standard Occupational Health Standard for Firefighters (GBZ 221) and reported normal or corrected‐to‐normal vision and no history of cardiovascular or neurological disorders. None had prior familiarity with the specific training facility. Written informed consent was obtained from all participants prior to data collection.

Participants were randomly assigned to strategy conditions (route‐strategy, *n* = 11; survey‐strategy, *n* = 11), stratified by expertise level.

### 2.2. Experimental Design

The study employed a 2 (Expertise: novice vs. expert) × 2 (Strategy: route vs. survey) between‐subjects quasi‐experimental design. Expertise was determined by operational experience and service duration. Navigation strategy was manipulated through differential task instructions designed to induce route‐based or survey‐based spatial representations (see Section 2.11).

### 2.3. Apparatus and Materials

#### 2.3.1. Task Environment

The experimental task was conducted in a CFBT facility—a three‐story structure designed for firefighter training in smoke behavior, fire dynamics, and structural search‐and‐rescue operations (Figure 1). The facility comprised nearly 30 interconnected rooms across three levels (total navigable area: 610 m^2^), featuring realistic architectural elements such as doorways, furniture, and structural obstacles. The facility was configured to create complete darkness, requiring participants to rely on handheld flashlights for navigation.

**FIGURE 1:**
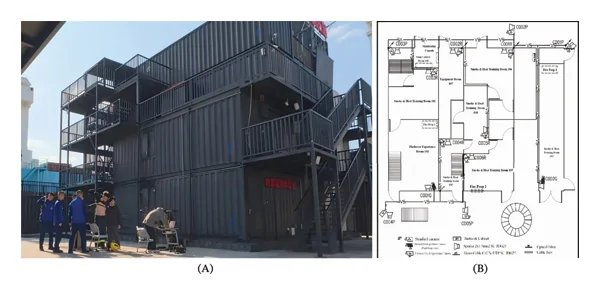
Experimental environment: CFBT facility configuration. (A) Exterior view of the CFBT facility located in Shanghai, China. (B) First‐floor layout showing the experimental navigation area.

Four adult‐sized training mannequins simulating victims were positioned throughout the facility (one on the first floor, two on the second floor, one on the third floor), selected to ensure comparable search difficulty across floors.

For the route‐strategy condition, nine fluorescent marker stickers (30 × 30 cm, yellow‐green, Figure 2) were affixed to walls along primary corridors, visible when proceeding along main navigation paths but not uniformly salient from all positions.

**FIGURE 2:**
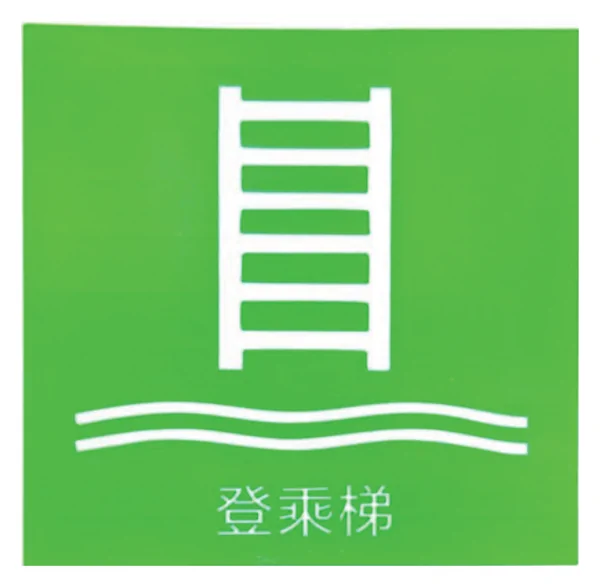
Fluorescent markers for route strategy induction.

### 2.4. Physiological Recording Equipment

#### 2.4.1. Cardiac Activity

Heart rate (HR) and HRV were continuously recorded using a Polar H10 chest‐strap HR sensor (sampling rate: 130 Hz [33]). The sensor was positioned below the pectoral muscles with the sensor unit centered on the sternum. Raw R‐R interval data were transmitted wirelessly to a smartphone running ECGLogger software (version 3.0) and stored in milliseconds for offline analysis.

To ensure high data quality during the vigorous physical activity, raw RR intervals were extracted and rigorously cleaned using the hrvanalysis library in Python. The automated artifact correction pipeline consisted of four sequential steps: (1) removal of nonphysiological outliers (RR intervals < 300 ms or > 1500 ms); (2) linear interpolation of the removed segments; (3) identification and removal of ectopic beats using the Malik rule; and (4) a final linear interpolation to yield a continuous time series of normal‐to‐normal (NN) intervals. Across all participants, the overall proportion of corrected or interpolated beats (including both movement‐induced artifacts and ectopic beats) was strictly monitored, averaging approximately 2.69% of the total recorded beats. This rigorous cleaning process ensured that the subsequent HRV metrics accurately reflected autonomic regulation rather than motion artifacts.

#### 2.4.2. Pupillary Responses

Pupil diameter was recorded using Tobii Pro Glasses 3 wearable eye‐tracking system (binocular tracking at 50 Hz; pupil measurement range: 2–8 mm; gaze accuracy: 0.6°). The lightweight head‐mounted system (total weight: 110 g) allowed secure fixation compatible with firefighter PPE, recording pupil diameter for both eyes with synchronized scene video.

### 2.5. Personal Protective Equipment (PEE) and Lighting

All participants wore standard firefighter PPE throughout the experiment, including turnout coat and trousers, helmet, gloves, boots, and self‐contained breathing apparatus (SCBA) cylinder (6.8 L; weight: 8.9 kg) worn on the back without face mask to allow unobstructed eye‐tracking (total PPE weight: approximately 16 kg). Participants carried a handheld LED flashlight (310 lumens; 15° beam angle) as their sole illumination source within the darkened facility (Figure 3).

**FIGURE 3:**
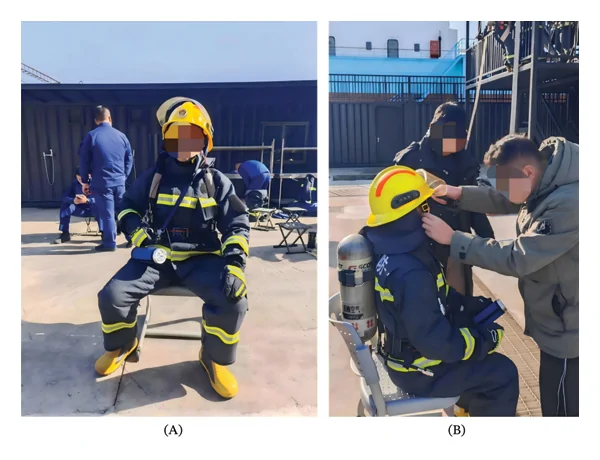
Participant equipment and physiological monitoring setup. Note. Facial features are obscured to protect participant anonymity. (A) Frontal view of participant equipped with full personal protective equipment, Tobii pro glasses 2 eye tracker, and polar H10 heart rate monitor. (B) Lateral view showing the self‐contained breathing apparatus (SCBA) carried on the participant’s back.

### 2.6. Supplementary Materials

#### 2.6.1. Walking Speed Assessment

A 50‐m straight pathway marked at start and end points, located outdoors adjacent to the CFBT facility.

### 2.7. Facility Floor Plan

A schematic 2D architectural plan showing all three floors with room boundaries and corridor connections, printed in black‐and‐white on A3 paper (297 × 420 mm), used for post‐task victim location marking in the survey‐strategy condition.

### 2.8. Marker Recognition Test

A set of 18 printed images (15 × 15 cm) comprising the nine fluorescent markers present in the facility (targets) and nine similar markers not present (foils), presented as a paper‐based recognition test in the route‐strategy condition.

### 2.9. Communication Equipment

Two‐way radio transceivers for verbal reporting of victim discoveries (one worn by participant, one operated by experimenter).

### 2.10. Timing Equipment

Digital stopwatch for recording walking speed and task completion time.

### 2.11. Procedure

Testing was conducted individually during morning hours (09:00–12:00). After providing informed consent, participants completed the following protocol (Figure 4):

**FIGURE 4:**
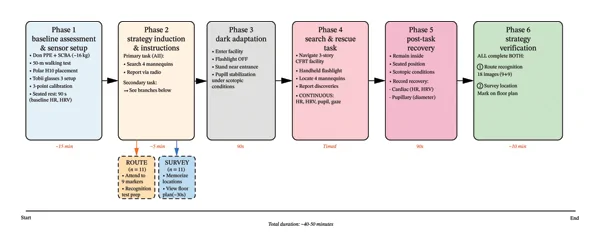
Schematic representation of the experimental protocol.

#### 2.11.1. Pre‐Task Preparation

Participants donned full firefighter PPE and completed a 50‐m walking speed test to establish baseline mobility. The Polar H10 HR sensor and Tobii Glasses 3 eye tracker were positioned and calibrated. Participants then rested quietly for 90 s while seated to establish resting baseline cardiac measures.

#### 2.11.2. Task Instructions and Strategy Manipulation

All participants received standardized instructions to search the darkened CFBT facility for training mannequins (simulated victims), report each discovery via radio, and exit when search was complete. The total number of mannequins (4 total: 1 on Floor 1, 2 on Floor 2, 1 on Floor 3) was not disclosed. Navigation strategy was manipulated through differential secondary task instructions: Route‐strategy condition (*n* = 11) participants attended to fluorescent marker stickers posted along corridor walls for subsequent recognition testing, directing attention toward sequential landmarks and encouraging route‐based spatial encoding. Survey‐strategy condition (*n* = 11) participants memorized mannequin locations for subsequent marking on a floor plan (shown for ∼30‐s pre‐task), encouraging survey‐based spatial representations.

This manipulation necessarily couples spatial strategy with secondary task characteristics: the route condition emphasizes visual attention to landmarks and recognition memory, whereas the survey condition emphasizes spatial working memory and map‐based recall. We view this coupling as ecologically valid, as real‐world navigation strategies are inherently tied to operational goals. All participants completed both post‐task assessments (marker recognition and location mapping) to verify manipulation effectiveness.

#### 2.11.3. Task Execution

Following 90‐s dark adaptation near the facility entrance (flashlight off), participants navigated freely through the three‐story facility using a handheld flashlight. During this phase, participants engaged in continuous, moderate‐to‐vigorous physical exertion. This involved alternating phases of walking, crouching, climbing stairs, and navigating structural obstacles while carrying approximately 16 kg of PPE. This intense physical activity induced a pronounced metabolic and cardiovascular load characteristic of real‐world firefighting. Task completion time was recorded from start to facility exit. Continuous cardiac and pupillary data were recorded throughout.

#### 2.11.4. Post‐Task Recovery

Immediately after task completion, participants remained seated near the entrance inside the darkened facility for 90 s while cardiac and pupillary recovery data were recorded under the same scotopic conditions as baseline to ensure comparable measurement conditions. This specific 90‐s duration was deliberately selected to ecologically simulate the extremely brief tactical pauses and rapid redeployment cycles typical of active search‐and‐rescue operations. In real‐world firefighting, extended 5‐min recovery periods are rarely feasible; thus, capturing the immediate, short‐term autonomic recovery provides a more operationally relevant metric of physiological resilience and readiness for subsequent tasks.

#### 2.11.5. Strategy Verification

Route condition participants completed a recognition test with 18 images (9 targets, 9 foils); survey condition participants marked victim locations on the floor plan. Accuracy was calculated as proportion correct (route) or proportion of victims correctly assigned to actual room locations (survey).

### 2.12. Dependent Measures

Physiological responses and recovery dynamics were quantified as ratio scores (task or recovery value/baseline value) to control for individual baseline differences [34]. All dependent measures are summarized in Table 2.

**TABLE 2:** Summary of dependent measures.

Measure	Description
Behavioral performance	
Completion time (s)	Task duration; lower indicates efficiency
Victims found	Count (max = 4); higher indicates effectiveness
Walking speed (m/s)	Baseline mobility (covariate)
Strategy verification	
Recognition accuracy (%)	Marker identification (route manipulation check)
Room location accuracy (%)	Victim placement (survey manipulation check)
Cardiac indices	
Baseline	HR (bpm), SDNN (ms), RMSSD (ms)
Task ratios	HRtask/HRbaseline, etc.; > 1.0 indicates increase
Recovery ratios	HRrecovery/HRbaseline, etc.; ⟶ 1.0 indicates recovery
Pupillary indices	
Baseline	Diameter (mm) under scotopic conditions
Task ratios	Diametertask/Diameterbaseline; > 1.0 indicates dilation
Recovery ratios	Diameterrecovery/Diameterbaseline; ⟶ 1.0 indicates recovery

*Note:* Cardiac data preprocessed using NeuroKit2 (Python); artifacts (> 20% deviation from 5‐interval median) corrected via cubic spline interpolation. b Pupillary data extracted using Tobii Pro Lab [35]; blinks (gaps ≤ 300 ms) linearly interpolated; binocular averaging applied.

### 2.13. Temporal Aggregation and Physiological Data Reduction

Because participants completed the search‐and‐rescue task in varying durations, raw physiological time series required temporal alignment for group‐level visualization and epoch‐based analysis. To enable overlay of individual trajectories and group‐level means despite differing task durations, we applied percentage‐based temporal normalization separately for each experimental phase (Baseline, Task, Recovery). For each participant: Individual task duration (time from start instruction to facility exit) was divided into 100 equally spaced time bins, each representing 1% of that participant’s task duration.

This percentage‐based temporal normalization allows all participants’ data to be plotted on a common proportional time axis regardless of absolute task duration differences. This approach preserves within‐phase temporal dynamics while enabling valid cross‐participant comparison.

Because task duration varied across participants (*M* = 8.7 min, SD = 1.4 min), phase‐averaged ratios were computed to standardize physiological changes relative to baseline: Task ratio = (mean during task)/(mean during baseline); Recovery ratio = (mean during recovery)/(mean during baseline).

### 2.14. Data Analysis

All analyses were conducted using SPSS 25.0 and Python 3.6.1. Significance threshold: *α* = 0.05 (two‐tailed), with Bonferroni corrections for multiple comparisons. Prior to main analyses, data were checked for normality (Shapiro–Wilk test) and homogeneity of variances. To address potential confounding effects identified in the literature, we initially planned an analysis of covariance (ANCOVA) to control for age and body mass index (BMI). However, in highly specialized populations such as firefighters, age is intrinsically linked to cumulative professional experience, creating inherent collinearity between the “expertise” grouping and “age” (i.e., experts are naturally older due to the time required to acquire expertise). Therefore, while age and BMI were explored as covariates to isolate physiological responses, the primary models (ANOVA) were retained to reflect the holistic nature of expertise. Both sets of analyses are interpreted with caution.

#### 2.14.1. Manipulation Check

Independent‐sample *t*‐tests compared strategy groups on recognition accuracy and location accuracy.

#### 2.14.2. Behavioral Performance

Task completion time was analyzed using 2 (Expertise: novice vs. expert) × 2 (Strategy: route vs. survey) ANCOVA with walking speed as covariate. Victims found was analyzed using 2 × 2 ANOVA.

#### 2.14.3. Physiological Responses

Separate 2 × 2 ANOVAs examined cardiac indices (HR, SDNN, RMSSD ratios) and pupillary indices (diameter ratios). Significant interactions were decomposed using simple effects tests.

#### 2.14.4. Recovery Dynamics

Recovery ratios (cardiac: HR, SDNN, RMSSD; pupillary: diameter) were analyzed using separate 2 × 2 ANOVAs.

Effect sizes reported as partial *η* ^2^ *p* (ANOVA) and Cohen’s *d* (pairwise comparisons). Additionally, sensitivity analyses using ANCOVA were performed to examine the potential confounding effects of age and BMI on all six cardiac indices. Bivariate Pearson correlations were calculated to explore the relationships between physiological metrics and behavioral performance.

Given the pilot nature of this study and the limited sample size (*N* = 22), a post hoc power analysis was conducted using G∗Power 3.1. For our primary analyses on task‐evoked HR increase (which showed a large effect size of partial *η* ^2^ *p* = 0.36), with alpha set at 0.05 and a total sample size of 22, the achieved statistical power (1 − β) was approximately 0.85. This indicates adequate power to detect the main effects observed. However, we acknowledge that the interaction effects and cross‐classified subgroup analyses inherently possess lower statistical power; thus, they remain strictly exploratory and require larger sample sizes for robust confirmation.

## 3. Results

### 3.1. Baseline Physiological Comparisons

To ensure that task‐related differences were not confounded by pre‐existing physiological variations, baseline measures were compared across groups using 2 (Expertise) × 2 (Strategy) ANOVAs. No significant main effects or interactions emerged for any cardiac measure (HR, SDNN, RMSSD) or pupillary measure (diameter; see Table 3 for descriptive statistics and omnibus tests). These null findings confirm baseline equivalence across experimental groups.

**TABLE 3:** Baseline physiological measures by expertise and strategy.

Measure	Novice‐survey	Novice‐route	Expert‐survey	Expert‐route	Overall model
*Cardiac measures*					
HR (bpm)	86.5 ± 10.2	90.3 ± 9.8	82.7 ± 13.5	91.2 ± 11.6	*F* (3,18) = 1.56, *p* = 0.237, *η* *p* ^2^ = 0.23
SDNN (ms)	91.4 ± 28.1	86.6 ± 30.5	90.6 ± 38.2	94.5 ± 32.7	*F* (3,18) = 2.17, *p* = 0.132, *η* *p* ^2^ = 0.29
RMSSD (ms)	48.3 ± 18.5	44.8 ± 22.7	54.9 ± 26.4	47.5 ± 24.1	*F* (3,18) = 1.54, *p* = 0.242, *η* *p* ^2^ ^*2*^ = 0.22

*Pupillary measure*					
Pupil (mm)	4.21 ± 0.58	4.35 ± 0.72	4.18 ± 0.65	4.29 ± 0.61	*F* (3,18) = 0.23, *p* = 0.877, *η* *p* ^2^ = 0.04

*Note:* Values are M ± SD. The Overall model column shows omnibus 2 × 2 ANOVA results. All group comparisons are nonsignificant.

### 3.2. Manipulation Check

Independent‐samples *t*‐tests confirmed successful strategy induction. The route‐strategy group showed significantly higher landmark recognition accuracy, whereas the survey‐strategy group showed significantly higher victim location accuracy (Table 4). This double dissociation confirms that the two groups engaged in qualitatively different forms of spatial processing.

**TABLE 4:** Manipulation check: performance differences between navigation strategy groups.

Measure	Orientation strategy		Route strategy					
	*M* (SD)	*n*	*M* (SD)	*n*	*t*	*df*	*p*	*d*
Landmark recognition	1.58 (1.09)	12	3.90 (2.47)	10	−2.78^∗^	11.43	0.017	1.22
Victim location identify	2.90 (1.29)	10	1.33 (0.89)	12	3.37^∗∗^	20	0.003	1.45

*Note:* Higher scores indicate better performance. The double dissociation pattern confirms successful manipulation of navigation strategies.

^∗^ *p* < 0.05.

^∗∗^ *p* < 0.01.

### 3.3. Behavioral Performance Analysis

#### 3.3.1. Task Completion Time

A 2 (Expertise) × 2 (Strategy) ANCOVA with walking speed as covariate revealed a significant main effect of strategy, *F* (1, 17) = 4.25, *p* = 0.047, *η* ^2^ *p* = 0.20, with the survey‐strategy group completing the task 1.42 min faster than the route‐strategy group, 95% CI [0.03, 2.88]. The main effect of expertise and the expertise × strategy interaction were not significant (Table 5). Walking speed did not predict completion time, *F* (1, 17) < 0.01, *p* = 0.940.

**TABLE 5:** Analysis of variance results for task performance.

Dependent variable	Source	df	*F*	*p*	*η* ^2^ *p*
Task completion time	Walking speed (covariate)	1, 17	0.006	0.940	0.000
	Expertise (A)	1, 17	0.040	0.843	0.002
	Navigation strategy (B)	1, 17	4.246	0.047^∗^	0.200
	A × B	1, 17	1.309	0.268	0.071
	Error	17			

Number of victims searched	Expertise (A)	1, 18	0.095	0.761	0.005
	Navigation strategy (B)	1, 18	0.095	0.761	0.005
	A × B	1, 18	0.095	0.761	0.005
	Error	18			

*Note:* ANCOVA controlling for walking speed.

#### 3.3.2. Number of Victims Searched

A 2 × 2 ANOVA revealed no significant main effects or interactions for the number of victims found (Expertise: *F* (1, 18) = 0.095, *p* = 0.761; Strategy: *F* (1, 18) = 0.095, *p* = 0.761; Interaction: *F* (1, 18) = 0.095, *p* = 0.761; see Table 5 and Figure 5), suggesting that this measure was not sensitive to the experimental manipulations.

**FIGURE 5:**
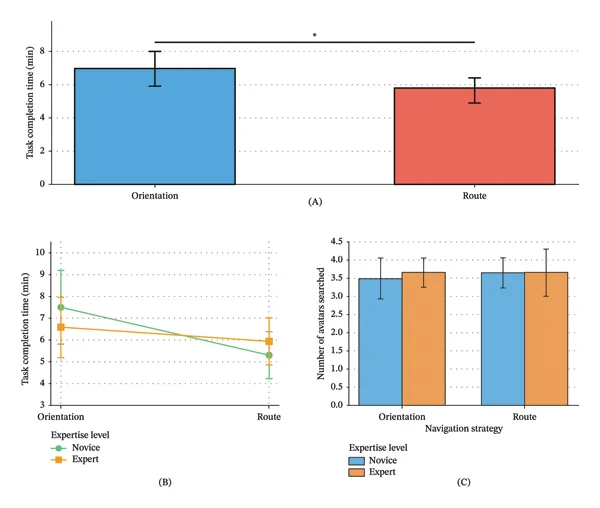
Comprehensive panel of behavioral performance results. Error bars represent 95% confidence intervals. ^∗^ *p* < 0.05; ns = not significant. (A) Main effect of navigation strategy (*p* = 0.047^∗^). (B) Expertise × strategy interaction (*p* = 0.268, ns). (C) Avatars searched (all *p* > 0.76, ns).

### 3.4. Physiological Responses

To examine psychophysiological responses to task demands, we analyzed cardiac and pupillary metrics across baseline, task, and recovery phases.

### 3.5. Temporal Dynamics of Cardiac and Pupillary Metrics

Figure 6 displays normalized time series of cardiac (HR, SDNN, RMSSD) and pupillary responses across experimental phases. Consistent with autonomic activation, HR increased and HRV metrics (SDNN, RMSSD) decreased during task performance, with gradual return toward baseline during recovery. Pupil diameter showed similar task‐related increases and post‐task recovery. Notably, the pupillary response showed less pronounced fluctuation compared to cardiac metrics, suggesting that cognitive workload remained relatively constant once participants engaged in the search task, whereas cardiovascular responses exhibited greater moment‐to‐moment variability. Statistical comparisons of phase‐averaged values are presented in Sections 3.7 and 3.8.

**FIGURE 6:**
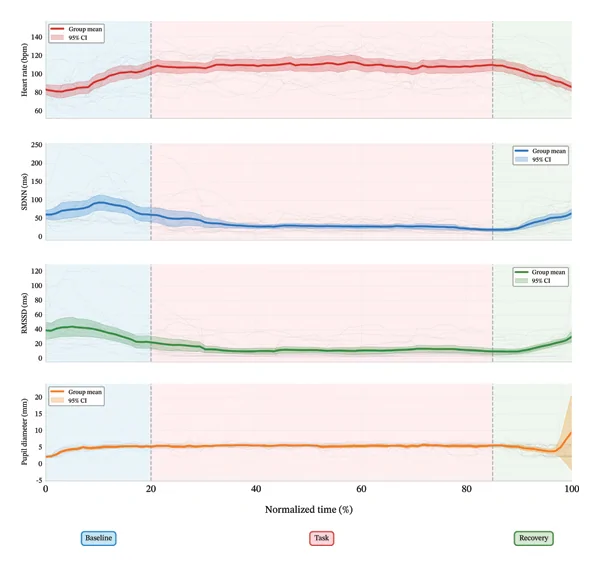
Temporal dynamics of cardiac and pupillary metrics across all participants. Note. Individual trajectories (gray) and group means with 95% CIs (colored) across baseline (0%–100%), task (0%–100%), and recovery (0%–100%) phases. Time normalized to phase duration.

Together, these multimodal physiological indicators provide converging evidence of task‐related autonomic activation and cognitive engagement. The coordinated changes in cardiac and pupillary measures underscore the integrative nature of physiological stress responses during high‐fidelity firefighting simulations.

### 3.6. Expertise and Navigation Strategy Effects on Physiological Responses

We conducted 2 (Expertise) × 2 (Strategy) ANOVAs on cardiac and pupillary metrics to examine task‐evoked changes and post‐task recovery patterns.

### 3.7. Cardiac Response Patterns

Prior to conducting ANOVAs, we examined the homogeneity of variance assumption using Levene’s tests for all cardiac and pupillary dependent variables. Results indicated that error variance equality was satisfied for all measures (see Table 6).

**TABLE 6:** Levene’s test for homogeneity of variance.

Dependent variable	Levene statistic	df1	df2	*p*	Assumption met
HR increase rate	2.537	3	18	0.093	Yes
SDNN decrease rate	0.180	3	18	0.908	Yes
RMSSD decrease rate	0.685	3	18	0.574	Yes
HR recovery rate	0.531	3	18	0.667	Yes
SDNN recovery rate	1.217	3	18	0.336	Yes
RMSSD recovery rate	1.186^a^	3	18	0.346	Yes
Pupil dilation rate	0.833	3	18	0.496	Yes
Pupil recovery rate	1.222	3	18	0.336	Yes

*Note:* All tests based on median except where noted.

^a^Median‐based test shown.

#### 3.7.1. HR Increase

A 2 × 2 ANOVA revealed significant main effects of expertise, *F* (1, 18) = 9.15, *p* = 0.008, *η* ^2^ *p* = 0.36, and strategy, *F* (1, 18) = 4.68, *p* = 0.049, *η* ^2^ *p* = 0.23, with no interaction (Table 7, Figure 7). Novices showed 20.0 percentage points greater HR elevation than experts, 95% CI [6.0%, 33.9%], *p* = 0.008, *d* = 0.95. Route‐strategy participants showed 14.3 percentage points greater HR increase than survey‐strategy participants, 95% CI [0.3%, 28.2%], *p* = 0.049, *d* = 0.68 (Table 8).

**TABLE 7:** Analysis of variance results for cardiac measures (task‐evoked changes).

Dependent variable	Source	SS	df	MS	*F*	*p*	*η* ^2^ *p*
HR increase rate	Expertise	0.195	1	0.195	9.15^∗∗^	0.008	0.364
	Strategy	0.100	1	0.100	4.68^∗^	0.049	0.226
	Expertise × Strategy	0.004	1	0.004	0.18	0.680	0.011
	Error	0.341	18	0.021			

SDNN decrease rate	Expertise	0.006	1	0.006	1.53	0.234	0.087
	Strategy	0.012	1	0.012	3.17	0.094	0.165
	Expertise × Strategy	< 0.001	1	< 0.001	< 0.01	0.946	< 0.001
	Error	0.060	18	0.004			

RMSSD decrease rate	Expertise	0.011	1	0.011	2.65	0.123	0.142
	Strategy	0.010	1	0.010	2.40	0.141	0.130
	Expertise × Strategy	0.004	1	0.004	1.08	0.314	0.063
	Error	0.064	18	0.004			

^∗^ *p* < 0.05.

^∗∗^ *p* < 0.01.

**FIGURE 7:**
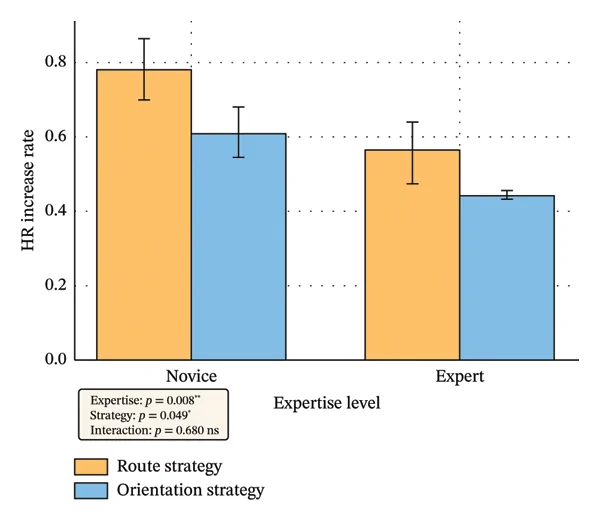
Heart rate response by expertise level and navigation strategy.

**TABLE 8:** Descriptive statistics for task‐evoked heart rate increase ratio by subgroups.

Measure	Group	*n*	*M*	SD	95% CI
HR increase rate	Novice–Survey	4	1.20	0.11	[1.02, 1.38]
	Novice–Route	6	1.46	0.14	[1.31, 1.61]
	Expert–Survey	6	1.21	0.04	[1.18, 1.25]
	Expert–Route	6	1.24	0.09	[1.15, 1.33]

*Note:* Values represent the ratio of task‐evoked heart rate to resting baseline heart rate (task HR/baseline HR). A value of 1.20 indicates a 20% increase from baseline.

Abbreviation: CI = confidence interval.

#### 3.7.2. HRV Suppression

Neither SDNN nor RMSSD decrease rates showed significant main effects or interactions (Table 7). For SDNN: expertise *F* (1, 18) = 1.53, *p* = 0.234, *η* ^2^ *p* = 0.09; strategy *F* (1, 18) = 3.17, *p* = 0.094, *η* ^2^ *p* = 0.17. For RMSSD: expertise *F* (1, 18) = 2.65, *p* = 0.123, *η* ^2^ *p* = 0.14; strategy *F* (1, 18) = 2.40, *p* = 0.141, *η* ^2^ *p* = 0.13.

### 3.8. Post‐Task Cardiac Recovery Patterns

#### 3.8.1. HR Recovery

Analysis revealed a significant Expertise × Strategy interaction, *F* (1, 18) = 7.08, *p* = 0.017, *η* ^2^ *p* = 0.31, with nonsignificant main effects (Table 9, Figure 8A).

**TABLE 9:** Analysis of variance results for cardiac measures (post‐task recovery).

Dependent variable	Source	SS	df	MS	*F*	*p*	*η* ^2^ *p*
HR recovery rate	Expertise	0.025	1	0.025	2.28	0.150	0.125
	Strategy	0.007	1	0.007	0.68	0.422	0.041
	Expertise × Strategy	0.077	1	0.077	7.08^∗^	0.017	0.307
	Error	0.174	18	0.011			

SDNN recovery rate	Expertise	0.014	1	0.014	0.16	0.697	0.010
	Strategy	0.093	1	0.093	1.05	0.320	0.062
	Expertise × Strategy	0.152	1	0.152	1.73	0.207	0.098
	Error	1.409	18	0.088			

RMSSD recovery rate	Expertise	1.052	1	1.052	4.67^∗^	0.042	0.226
	Strategy	0.307	1	0.307	1.36	0.182	0.078
	Expertise × Strategy	1.071	1	1.071	4.76^∗^	0.044	0.229
	Error	3.603	18	0.225			

**FIGURE 8:**
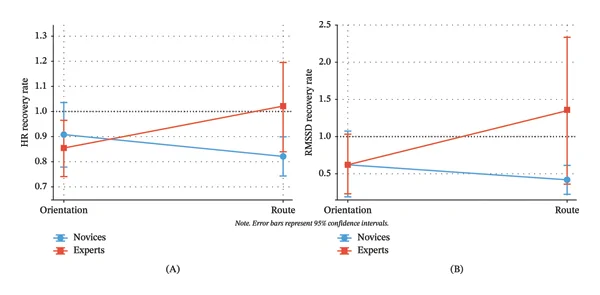
Expertise × strategy interaction for cardiac recovery. (A) Heart rate recovery. (B) RMSSD recovery.

#### 3.8.2. SDNN Recovery

No significant effects emerged: expertise *F* (1, 18) = 0.16, *p* = 0.697, *η* ^2^ *p* = 0.01; strategy *F* (1, 18) = 1.05, *p* = 0.320, *η* ^2^ *p* = 0.06; interaction *F* (1, 18) = 1.73, *p* = 0.207, *η* ^2^ *p* = 0.10 (Table 9).

#### 3.8.3. RMSSD Recovery

A significant main effect of expertise, *F* (1, 18) = 4.67, *p* = 0.042, *η* ^2^ *p* = 0.23, and Expertise × Strategy interaction, *F* (1, 18) = 4.76, *p* = 0.044, *η* ^2^ *p* = 0.23, emerged (Table 9, Figure 9; Figure 8B). Experts showed 46.3 percentage points greater RMSSD recovery than novices, 95% *CI* [0.9%, 91.8%], *p* = 0.046, *d* = 0.68.

**FIGURE 9:**
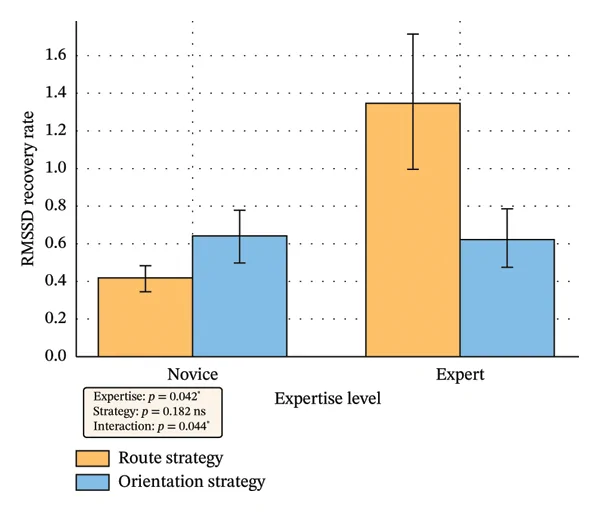
RMSSD recovery by expertise level and navigation strategy.

#### 3.8.4. Simple Effects Decomposition

For both HR and RMSSD recovery, simple effects revealed crossover interactions (Table 10): within route‐strategy conditions, experts showed significantly better recovery than novices (HR: Δ = 19.6 pp, *p* = 0.019, *d* = 1.81; RMSSD: Δ = 93.1 pp, *p* = 0.038, *d* = 1.74, Table 11, Figure 8.), whereas no expertise differences emerged within survey‐strategy conditions for either HR recovery (*p* = 0.369) or RMSSD recovery (*p* = 0.983).

**TABLE 10:** Descriptive statistics for cardiac recovery rates by expertise and strategy.

Measure	Group	*n*	*M*	SD	95% CI
HR recovery rate	Novice–Survey	4	0.910	0.079	[0.784, 1.035]
	Novice–Route	5	0.823	0.061	[0.747, 0.899]
	Expert–Survey	6	0.855	0.098	[0.753, 0.958]
	Expert–Route	5	1.019	0.145	[0.839, 1.200]

RMSSD recovery rate	Novice–Survey	4	0.635	0.265	[0.214, 1.055]
	Novice–Route	5	0.417	0.164	[0.214, 0.621]
	Expert–Survey	6	0.631	0.369	[0.243, 1.018]
	Expert–Route	5	1.348	0.757	[0.409, 2.287]

*Note:* Values closer to 1.0 indicate more complete cardiovascular restoration; values > 1.0 indicate parasympathetic rebound exceeding baseline.

**TABLE 11:** Summary of simple effects tests for cardiac recovery interactions.

Comparison	HR recovery			RMSSD recovery		
	*t*	*p*	*d*	*t*	*p*	*d*
Within‐strategy expertise comparisons						
Survey: Novice vs. Expert	0.95	0.369	0.63	0.02	0.983	0.01
Route: Novice vs. Expert	−3.17	0.019^∗^	1.81	−2.88	0.038^†^	1.74
Within‐expertise strategy comparisons						
Novice: Survey vs. Route	1.95	0.092	1.25	1.54	0.168	1.00
Expert: Survey vs. Route	−2.50	0.034^†^	1.35	−2.24	0.069	1.21

*Note:* Negative *t*‐values indicate higher values for experts or route strategies.

^†^ *p* < 0.05 but > 0.025 (marginal after correction).

^∗^ *p* < 0.025 (survives Bonferroni correction).

#### 3.8.5. Covariate Analysis for Cardiac Metrics

To rigorously address potential confounding variables identified in the literature, a sensitivity analysis using ANCOVA was conducted with age and BMI included as covariates across all six cardiac dependent variables (task‐evoked changes and post‐task recovery rates for HR, SDNN, and RMSSD). When adjusting for age and BMI, the previously observed significant main effects and interactions on task‐evoked HR increase, post‐task HR recovery, and RMSSD recovery were attenuated to nonsignificance (e.g., the expertise main effect on HR increase: *F* (1, 16) = 1.12, *p* = 0.306; HR recovery: *F* (1, 16) = 1.05, *p* = 0.321; RMSSD recovery: *F* (1, 16) = 0.943, *p* = 0.347, *η* ^2^ *p* = 0.059). Similarly, the nonsignificant findings for SDNN and task‐evoked HRV suppression remained nonsignificant. This statistical attenuation suggests an inherent collinearity between chronological age and professional expertise in the firefighting population. Essentially, the physiological regulatory advantages attributed to “expertise” are inextricably linked to the chronological maturation and years of service required to attain expert status. Consequently, statistically partialing out age inadvertently removes the natural demographic variance essential to the expert profile. Therefore, the primary ANOVA models are retained as the most ecologically valid representation of expertise in this specialized occupational group.

### 3.9. Pupillary Response and Recovery Patterns

Pupillary metrics showed no significant effects. For task‐evoked dilation: expertise *F* < 0.01, *p* = 0.979; strategy *F* = 0.01, *p* = 0.935; interaction *F* = 2.17, *p* = 0.161. For pupil recovery: expertise *F* = 1.02, *p* = 0.328; strategy *F* = 0.01, *p* = 0.918; interaction *F* = 1.41, *p* = 0.254 (Table 12). These null findings contrast sharply with the robust cardiac differentiation observed (Sections 3.7 and 3.8), likely reflecting methodological constraints of pupillometry in operational field settings characterized by uncontrolled lighting, dynamic head movements, and extreme ambient conditions (discussed in Section 4).

**TABLE 12:** Analysis of variance results for pupil measures.

Dependent variable	Source	SS	df	MS	*F*	*p*	*η* ^2^ *p*
Task‐evoked pupil dilation	Expertise	0.441	1	0.441	< 0.01	0.979	< 0.001
	Strategy	4.122	1	4.122	0.01	0.935	< 0.001
	Expertise × Strategy	1308.724	1	1308.724	2.17	0.161	0.126
	Error	9042.212	18	602.814			

Post‐task pupil recovery	Expertise	536.620	1	536.620	1.02	0.328	0.064
	Strategy	5.774	1	5.774	0.01	0.918	0.001
	Expertise × Strategy	739.492	1	739.492	1.41	0.254	0.086
	Error	7890.082	18	526.005			

### 3.10. Correlations Between Physiological Metrics and Task Performance

To explore the relationship between internal physiological load and overt external performance, bivariate Pearson correlation analyses were conducted. All six cardiac indices (task and recovery rates for HR, SDNN, RMSSD) and pupillary metrics were correlated with task completion time and the number of victims located. Results indicated a decoupling between physiological metrics and behavioral outcomes, as no significant linear correlations emerged. For instance, post‐task RMSSD recovery rate—which strongly differentiated experts from novices—showed no significant association with task completion time (*r* = −0.167, *p* = 0.481, 95% CI [−0.568, 0.297]) or the number of victims located (*r* = 0.375, *p* = 0.103, 95% CI [−0.081, 0.701]). Task‐evoked HR increase similarly did not predict task duration (*r* = −0.109, *p* = 0.646, 95% CI [−0.526, 0.350]). Furthermore, pupillary metrics were not significantly correlated with any behavioral outcomes (e.g., pupil dilation and completion time: *r* = 0.142, *p* = 0.528; pupil recovery and victims found: *r* = −0.086, *p* = 0.704). This physiological‐performance decoupling suggests that within the moderate‐to‐high demands of this simulation, firefighters maintained external performance via adaptive effort allocation, absorbing the costs internally rather than allowing overt performance to degrade.

## 4. Discussion

The present study investigated physiological efficiency as a hallmark of firefighter expertise during search‐and‐rescue operations. Expert and novice firefighters achieved equivalent task performance—similar completion times and victim identification—yet differed profoundly in physiological costs. This dissociation provides preliminary evidence that expertise manifests not only in what is accomplished, but in how efficiently it is accomplished [36, 37].

Three key findings emerged. First, experts exhibited 20% lower cardiovascular activation during task execution despite equivalent performance, supporting physiological efficiency (H1a partially supported). Second, experts demonstrated 46% superior parasympathetic recovery post‐task, particularly when employing route‐based strategies (H1b and H3 supported). Third, cardiac measures successfully differentiated expertise and strategy effects, whereas pupillary responses showed uniform elevation across participants, revealing methodological insights about multimodal assessment in operational environments.

Our finding that experts and novices achieved equivalent task performance yet differed substantially in cardiovascular activation challenges traditional performance‐centric definitions of expertise [1, 38]. This pattern is consistent with emerging frameworks emphasizing efficiency over outcomes [39] and aligns with recent work showing that experts can “do more with less” physiologically across domains [40, 41]. The 20% reduction in HR elevation observed in experts represents substantial cardiovascular economy, comparable to expertise differences reported in police firearms training [42], surgical procedures [43], and emergency flight simulations [44]. This convergence suggests physiological efficiency may be a domain‐general signature of expertise, reflecting fundamental differences in regulatory resource allocation during complex performance. Critically, this efficiency advantage emerged despite experts and novices wearing identical PPE (16 kg) and navigating the same environmental conditions (complete darkness, time pressure). This rules out confounds related to physical fitness or equipment familiarity and isolates cognitive‐affective efficiency as the primary differentiator. Experts appear to have developed more efficient cognitive strategies, reduced threat appraisal, or greater emotional regulation—factors that collectively reduce sympathetic activation even during objectively demanding tasks [12, 13].

The absence of expertise differences in behavioral performance—task completion time (*d* = 0.33, *p* = 0.843) and victim identification (*d* = 0.33, *p* = 0.761)—requires interpretation. This dissociation was further corroborated by our correlation analyses, which revealed a complete decoupling between physiological indices and behavioral outcomes. We propose that this reflects a phenomenon known as “physiological‐performance decoupling” [34], highlighting the sensitivity limits of outcome‐based metrics. Task completion time and accuracy capture only final products, not process efficiency. Two individuals completing identical searches may follow radically different paths: one navigating fluidly with minimal cognitive deliberation and low autonomic arousal, another advancing haltingly with continuous error correction and high sympathetic activation. Our data suggest precisely this scenario—experts and novices converged on similar behavioral endpoints through divergent underlying mechanisms. When task demands are high but manageable, individuals can maintain stable external performance by compensating through increased internal mental effort and sympathetic drive. Novices mask their less efficient strategies by “absorbing” the physiological costs, driving up their cardiovascular activation to keep pace with experts. Experts, conversely, achieve the same outcomes with significantly lower autonomic costs due to superior stress regulation. This lack of linear correlation emphasizes exactly why physiological monitoring is crucial: relying solely on performance metrics creates a dangerous blind spot, failing to identify personnel who are maintaining overt performance but operating near the edge of physiological exhaustion.

From an operational perspective, physiological efficiency may be more consequential than single‐task performance for predicting real‐world effectiveness. Firefighting involves extended operations (multihour incidents), sequential demands (repeated searches, rescues, equipment operations), cumulative physiological strain (heat stress, exertion, sleep deprivation), and unpredictable critical events requiring reserve capacity. The firefighter who completes routine searches with 20% lower cardiovascular activation possesses critical advantages: reduced cumulative allostatic load across repeated exposures, greater physiological headroom to respond to sudden critical events (flashover, structural collapse, victim extraction), faster intertask recovery enabling sustained effectiveness, and lower long‐term cardiovascular morbidity risk. These benefits accrue precisely because experts conserve physiological resources during routine operations—a capability invisible to single‐task behavioral metrics but central to sustained operational effectiveness and career longevity.

Perhaps the most theoretically significant finding is the crossover interaction between expertise and navigation strategy in cardiac recovery patterns (H3 supported). Experts demonstrated superior recovery specifically when using route‐based strategies (102% HR recovery, 135% RMSSD recovery), whereas novices showed no strategy‐related differences. This pattern suggests that expertise does not uniformly enhance physiological efficiency across all task approaches; rather, experts benefit selectively from strategies that align with their training and procedural knowledge. This finding has important implications for understanding firefighting expertise. Route‐based navigation—relying on sequential landmark recognition and procedural turn sequences—maps directly onto standard firefighting search patterns taught during training (e.g., “right‐hand search,” systematic wall‐following protocols; [11, 45]). For experts, these proceduralized strategies may have become automated through extensive practice, requiring minimal executive control and enabling rapid physiological down‐regulation post‐task [46]. In contrast, survey‐based strategies requiring allocentric spatial mapping may impose continued cognitive load even for experts, as maintaining survey representations in dark, claustrophobic environments exceeds the ecological validity of typical training scenarios.

The strategy‐expertise interaction has direct implications for training design. Current firefighter training emphasizes proceduralized route‐based search patterns, which our data suggest are physiologically efficient—but only for trained personnel. For recruits early in training, prescribing rigid route‐based protocols may impose excessive strain. A phased approach—beginning with survey‐based spatial awareness and transitioning to proceduralized routes as skills consolidate—may optimize learning efficiency and physiological adaptation. Critically, training should cultivate both procedural mastery and adaptive flexibility, avoiding over‐reliance on rigid protocols that may fail in atypical scenarios.

An unexpected finding was the complete absence of expertise or strategy effects on pupillary measures, despite robust cardiac differentiation. While laboratory research demonstrates that pupil diameter reliably tracks cognitive load [26, 47], our operational environment introduced multiple confounds. Extreme sustained cognitive‐perceptual demands may have imposed ceiling effects on sympathetic arousal, maximally dilating pupils across all participants [48]. Additionally, handheld flashlights created continuous unpredictable fluctuations in retinal light exposure, activating pupillary light reflexes that obscured cognitive load signals [49], while physical exertion from PPE (16 kg) likely elevated systemic arousal uniformly, reducing between‐group variance.

Critically, cardiac measures proved far more robust to these environmental challenges, with HRV indices successfully differentiating expertise despite identical stressors. This suggests that vagal tone modulation is less susceptible to momentary physical and environmental artifacts than pupillary sympathetic responses. For applied research in operational settings, cardiac monitoring may offer superior ecological validity when environmental control is limited. Future field studies should prioritize cardiac and electrodermal measures in uncontrolled environments, or implement head‐mounted constant‐output lighting to minimize pupillary light‐reflex artifacts.

A critical methodological insight from our study concerns the inherent collinearity between chronological age and professional expertise, as revealed by our covariate sensitivity analyses. When Age and BMI were statistically controlled, the robust cardiovascular advantages observed in experts were attenuated to nonsignificance. Rather than invalidating the main effects, this attenuation highlights a fundamental reality of ecologically valid occupational research: true operational expertise is inextricably bound to the years of service, physical maturation, and biological aging required to attain it. In highly specialized populations like firefighters, statistically isolating “age” inadvertently strips away the temporal accumulation of experience, procedural memory consolidation, and stress‐inoculation that define the expert profile. Therefore, the physiological adaptations we observed should be interpreted holistically as the product of longitudinal professional maturation. Future research in occupational settings must carefully weigh the mathematical appeal of covariate adjustment against the ecological risk of over‐correcting natural demographic variance that is intrinsic to the construct of expertise itself.

### 4.1. Practical Implications

Our findings have direct applications for firefighter training and operational protocols in three domains: expertise assessment, fatigue management, and strategy instruction.

#### 4.1.1. Physiological Monitoring in Training and Operations

Current firefighter competency evaluations focus primarily on task completion time and procedural accuracy (NFPA 1001; [8]). Our data suggest these metrics miss a critical dimension: physiological efficiency. Two firefighters completing identical searches may differ substantially in cardiovascular strain and recovery capacity—differences that become critical during extended operations or multialarm incidents. Incorporating physiological monitoring into training assessments could identify individuals who, despite adequate behavioral performance, exhibit excessive autonomic activation suggesting incomplete skill consolidation. Portable HR monitors are now sufficiently affordable and validated for operational use [33, 50], making routine cardiac assessment feasible in training contexts.

#### 4.1.2. Recovery Capacity Has Particular Operational Significance

The dramatic differences in RMSSD recovery between experts and novices (46% overall advantage; 93% points in route‐strategy conditions) highlight vagal restoration as a key expertise marker. Incomplete recovery compounds across repeated exposures: firefighters with poor parasympathetic restoration accumulate physiological debt, increasing injury risk and decision‐making errors [16]. Simple HRV monitoring during rest periods in multialarm incidents could identify personnel requiring extended recovery before reassignment, optimizing crew rotation strategies.

#### 4.1.3. Strategy‐Matched Training Progressions

The expertise × strategy interaction suggests that optimal training approaches may be skill‐dependent. Training curricula could implement phased strategy instruction: novice recruits might initially emphasize spatial awareness and flexible problem‐solving, transitioning to proceduralized route‐based protocols as skills consolidate. Critically, training should cultivate both procedural mastery and adaptive flexibility, avoiding over‐reliance on rigid protocols that may fail in atypical scenarios.

## 5. Limitations and Future Directions

Several limitations must be acknowledged. First, the relatively small sample size (*n* = 22) and the exclusive inclusion of male participants limit statistical power and broad generalizability, though this reflects the demographic reality of frontline combatants in our region and the logistical constraints of high‐fidelity live‐fire facilities. Future larger‐scale studies are required to confirm these preliminary findings. Second, due to the highly ambulatory nature of the task, our HRV analysis was restricted to time‐domain indices (RMSSD, SDNN). Future research should incorporate frequency‐domain (particularly considering the ongoing debate regarding the physiological significance of the LF/HF ratio) and nonlinear HRV parameters (e.g., DFA, SD1/SD2) to capture autonomic complexity more comprehensively. These advanced metrics have been shown to provide a more comprehensive characterization of autonomic dynamics and translational clinical value [13, 27, 51–53]. Third, while operational routines were standardized, unmeasured confounding factors—such as individual baseline fitness, prior sleep quality, and moment‐to‐moment respiratory patterns under the heavy SCBA—could influence autonomic responses. Lastly, subjective cognitive load questionnaires were omitted to maintain strict ecological immersion. Future studies utilizing advanced smart‐garment technologies may better capture continuous respiratory and subjective data without disrupting operational flow.

## 6. Conclusion

In summary, this study provides preliminary evidence that firefighter expertise manifests as physiological efficiency rather than solely performance outcomes. Despite equivalent task completion, experts demonstrated substantially lower cardiovascular activation and superior parasympathetic recovery compared to novices, with these advantages systematically modulated by navigation strategy. These findings advance theoretical understanding of expertise as a multidimensional construct encompassing not only performance capacity but also regulatory efficiency—the ability to achieve task goals while minimizing physiological costs and maximizing recovery capacity. Practically, the advent of portable cardiac monitoring technology now enables integration of physiological assessment into training and operational protocols, allowing real‐time identification of individuals requiring additional skill development or recovery time. This approach supports both performance optimization and long‐term occupational health in high‐stakes domains where sustained physical and cognitive demands are routine.

## Funding

This study was financially supported by Shanghai Fire Research Institute of MEM, Shanghai Fire Research Institute of MEM 25SX45, and China National Fire and Rescue Administration, 2024XFCX33.

## Disclosure

A preprint version of this manuscript has been deposited on Authorea and is available at https://www.authorea.com/users/1025477/articles/1385269-math_shortcuts-cardiac-efficiency-as-a-marker-of-firefighter-expertise-heart-rate-variability-monitoring-in-realistic-search-and-rescue-training?commit=b8d994288a000b55972960071af95817c077a991.

## Conflicts of Interest

The authors declare no conflicts of interest.

## Data Availability

The data that support the findings of this study are available from the corresponding author upon reasonable request.
